# Evaluation of QoL in neurofibromatosis patients: a systematic review and meta-analysis study

**DOI:** 10.1186/s12883-019-1338-y

**Published:** 2019-06-12

**Authors:** Akram Sanagoo, Leila Jouybari, Fatemeh Koohi, Fatemeh Sayehmiri

**Affiliations:** 1 Nursing Research Center, Goletsan University of Medical Sciences, Gorgan, Iran; 2 Department of Epidemiology, School of Public Health, Birjand University of Medical Sciences, Birjand, South Khorasan Province Iran; 3 Student Research Committee, Neuroscience Research Center, Shahid Beheshti University of Medical Sciences, Tehran, Iran

**Keywords:** Neurofibromatosis, Meta-analysis, Quality of life, Schwannomatosis

## Abstract

**Background:**

The neurofibromatoses (NF) are a group of genetic disorders that interfere negatively with the quality of life (QoL) and influence physical, emotional and social statuses. Studying the effects of neurofibromatoses on various aspects of QoL seems important to implement beneficial strategies in increasing the QoL of NF patients. The aim of this study was to review the literature on quality of life in patients with NF and quantitatively evaluate the effects of Neurofibromatosis on various aspects of quality of life by synthesizing available studies.

**Methods:**

This article is written according to the PRISMA checklist. Different databases including PubMed, Scopus, Google scholar and Web of Science were searched to identify studies that examined QoL of patients with neurofibromatosis. The relevant data obtained from these papers were analyzed by a random-effects model. The heterogeneity of studies was calculated using the I^2^ index and Egger test was used to determine publication bias. The information was analyzed by R and STATA Ver 14.

**Results:**

Twelve studies were selected as eligible for this research and were included in the final analysis. The number of participants in the study was 7314 individuals containing 910 NF patients (642 NF1 and 268 NF2) and 6404 healthy subjects. The mean scores of sub-scales of QoL were significantly lower in NF patients compared with control except for the scale of cohesion. Family and NF patients had lower quality of life in all aspects of QoL than controls. Also, this meta-analysis shows that NF negatively effects on physical function, bodily pain, mental health, social function and general health. Subgroup analysis showed that NF had negative effects on all sub-scales of QoL if the study was conducted in adults and used a SF-36 questionnaire.

**Conclusion:**

This meta-analysis suggests that NF is a broad spectrum disease, affecting physical function, bodily pain, mental health, social function and general health.

## Background

Neurofibromatosis is a group of inherited genetic disorders (NF1, NF2, and Schwannomatosis) which affects the peripheral and central nervous system; it predisposes affected individuals to develop tumors in the nerve’s sheath across the body; leads to hearing loss, facial weakness, poor gait; and causes chronic pain [1, 2]. NF1 is a dominant autosomal disease which is the most common of the neurofibromatoses (typically 1 in 3500 births) and has manifestations such as skin lesions, involvement of the central nervous system, skeletal lesions and effects on the endocrine glands [3, 4]. The preliminary symptoms of the disease are skin lesions in the form of Café au lait spots; this type of neurofibromatosis is also known by the name of Von Recklinghausen disease [5, 6]. Other manifestations including pigmentary abnormalities, low-grade gliosis, skeletal dysplasia, and the involvement of numerous other organ systems are seen in NF1 patients [3–6]. Many of the serious complications of NF1 including orthopedics problems, disfiguring plexiform neurofibromas, optic pathway gliosis, renal artery stenosis and cognitive impairment occur in childhood [7]. Type 2 neurofibromatosis (NF2) is also an autosomal dominant disorder which has a lower prevalence (1 in 25,000) [8, 9]. It is characterized by the predisposition to develop multiple tumors including spinal cord gliosis, neurofibromas, meningioma, and schwannomas of the cranial nerves or spinal nerves [9, 10]. Tumors of the cochlear nerve in this disease can lead to loss of hearing, dizziness and vertigo, significant ataxia, equilibrium disorders, headache, tinnitus, pain and dysfunction of the facial muscles [2, 9, 10]. Patients exhibit this disease during the teenage years or second decade of their life [8]. NF1, NF2 and their various physical, cognitive, and social complications can cause a significant decrease in quality of life of affected individuals [3–10].

Quality of life is presented as an index of all factors effecting the quality of life of the patient to map the disease’s progress [11, 12]. Health related quality of life (HRQoL) is a concept which is specifically related to personal health and measures the function, welfare and general understanding of the patient in each one of these three aspects, physical, psychological and social [11]. It is possible to examine the effect of the illness on QoL through HRQoL and questionnaires specific to the disease or general questionnaires [13]. In most studies a general health questionnaire (the short form 36 health survey: SF-36) and a questionnaire more specific to QoL (Skindex-29) are used to measure QoL [14, 15]. The short form 36 health survey (SF-36) has been designed to survey health status in clinical practice, research, health policy evaluations and general population surveys [14]. Skindex is a QoL questionnaire used to analyze QoL in dermal diseases [15].

Due to the clinical implications of neurofibromatosis and their negative effects on quality of life, planning of prevention and treatment of this disease seems necessary [6–9]. The majority of the care models for NF are biomedical; whereas there has been an increased awareness of the mind–body connection in quality of life in these patients [1, 16]. Thus evaluation of symptoms and complications related to NF seems important in order to implement beneficial strategies about QoL in this population [17, 18]. So, research has started to assess quality of life in this population, and many studies in a broad variety of populations have been conducted on this topic, yielding different outcomes. Some studies demonstrated that NF has a negative effect on the QoL physical, emotional, psychosocial and social statuses, whereas others have reported controversial data [19–28]. Moreover, with this large body of literature, an overall estimation of its association is important, as is indicated by the breadth and quality of these studies. In order to authenticate the conducted studies and as a way of synthesizing their findings, performing a meta-analysis appears necessary [29].

Previously, two systematic reviews have explored the existing data on the effects of NF on the quality of life; a 2013 review study reviewed the literature on quality of life among adult patients with neurofibromatoses and showed that adult patients with NF1, NF2 and schwannomatosis suffer from impaired QoL [30]. Another systematic review in 2015 on children and adolescents with NF reported that these patients have lower general QoL compared to population norms [31]. Although these studies reviewed the association of NF and QoL, and reported that such association indeed exists, the association of neurofibromatosis and QoL has not been quantitatively evaluated, and our knowledge about the clinical importance and implications of this association is still limited. Since a combination of different studies via meta-analysis leads to a suitable sample size and better resolution, it can provide an overall precise and valid understanding of a desired subject relative to the separated reported studies [29]. So it seems that assessment of the effects of NF on QoL via meta-analysis is a useful tool that results in an overall and clear understanding of this disease. This meta-analysis was performed to quantitatively evaluate the effects of Neurofibromatosis (NFT) on various aspects of quality of life by synthesizing available studies.

## Methods

### Search methods *for identification* of studies

This meta-analysis was done on the basis of the Preferred Reporting Items for Systematic Reviews and Meta-Analyses (PRISMA) criteria to identify, select, and determine eligibility of studies for inclusion in the study [32]. Two authors independently performed a formal computer-assisted search in the Scopus, the Institute for Scientific Information Web of Science, PubMed, EmBase, and Cochrane Library databases for relevant articles published up to August 2017. Searching was done using the terms neurofibromatosis, NF1, NF2 and schwannomatosis in combination with the following key terms such as quality of life, QoL and health related quality of life, well-being. The search scope was developed using the symbol ‘*‘and an advanced search of words or statements created by Boolean operators was performed. The meta-analysis was limited to studies published in English. The list of recognized articles was scanned, and the reference lists of all related reviews and main articles were searched manually for more references. The studies obtained through the search strategy and other references were entered with the reference management software (Endnote), and duplicate items were deleted using the features of this software.

### Selection of studies

#### Inclusion and exclusion criteria

**Fig. 1:**
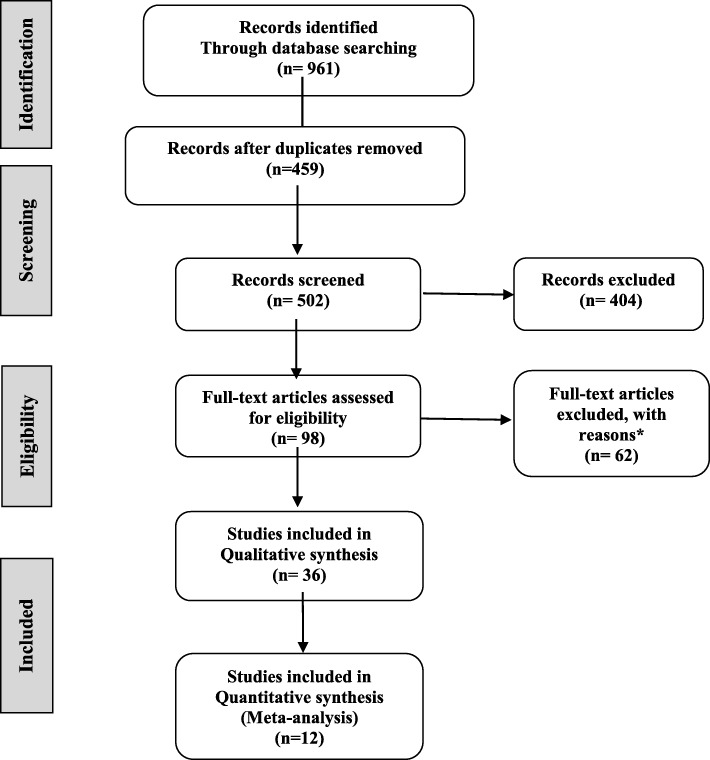
Prisma flow diagram illustrating selection of articles

In this stage, titles and abstracts of studies were reviewed and probable related studies were identified. In the next stage, the final studies were selected by referring to the full text of the remaining studies and based on the entry criteria (Fig. 1). All studies conducted on the quality of life among children, adolescents and adult patients with neurofibromatosis (NF1, NF2 and schwannomatosis) and used standard criteria for measuring the quality of life [SF-36 [19–21, 24, 25, 27, 28], CHQ-PF50 [22], (ITQOL [23], and CHQ [26]] in these patients were included in the study. Studies that discussed the quality of life of patients with neurofibromatosis, but the average quality of life was not reported in them, or studies with inadequate data or studies in which access to the text was not possible, were excluded from the meta-analysis. Meta-analyses or systematic considerations were also excluded.

### Data extraction

For each study, the extracted data includes the year and place of the study, the author, the number of participants in the study, the average age of the participants, the type of questionnaire used, and the mean of different dimensions of quality of life and their standard deviation. These information and data were entered in a standard data extraction form and finally into Microsoft Excel.

### Evaluation of the quality of selected studies

The quality of studies was evaluated using the Newcastle–Ottawa Scale checklist [33] (Table 1). The (NOS: Newcastle–Ottawa Scale) ranges from zero to nine stars. Selected papers were ranked in three groups according to NOS quality assessment: 1- Low quality (up to 3 stars), 2- Medium quality (4–6 stars) and 3- High quality (more than seven stars).

**Table 1:** Quality assessment of included articles according to the Newcastle–Ottawa Scale checklist

Researchers	Selection				Comparability		Exposure		
	1	2	3	4	A	B	1	2	3
Page. PZ [19]	*	*	*	*				*	*
Kodra. Y [20]	*	*						*	*
Merker. VL [21]	*	*	*	*	*			*	*
Vardarinos. A [22]	*	*	*	*			*	*	
Ostenbrink. R [23]	*	*	*	*	*			*	*
Wolkenstein. P [24]	*		*		*			*	*
Hornigold. RE [25]	*	*	*	*	*	*		*	*
Krab. LC [26]		*	*					*	*
Hornigold. RE [27]	*	*			*		*	*	
Merker. VL [28]		*		*				*	*

* Has a quality score

The criteria considered to measure bias in the authors’ intended studies included: reference to the time and place of the study, describing exit and entry criteria of the participants, how to measure the variables, the statistical methods used and preparing reports on standard deviation or confidence intervals of the estimates.

### Data analysis

Data was classified according to the type of questionnaire used. A combined estimate of the mean of quality of life was calculated for the different dimensions of quality of life with studying the effect and the same questionnaire. To assess heterogeneity between the studies, the *chi*-*squared test* for the 10% confidence level (for evaluation of heterogeneity between studies statistically; P less than 0.01) and I^2^ index (to evaluate the heterogeneity of the results) was used. The random effects model or the fixed effect model was used to analyze the data, according to the heterogeneity between the studies; when the results of studies were heterogeneous, the analysis was performed using a random-effects model and wherever there was no heterogeneity for the outcome, the fixed effects model was used to pool analysis Integrated estimations and the related confidence interval of 95% were evaluated using forest plots as visuals. The Begg test was used *for evaluating* publication bias. *P* values < 0.05 were considered significant in heterogeneity tests. Statistical analyses were carried out using R software (version 3.2.1) and STATA (version 11.1).

## Results

### Description of the studies

In the present study, a total of 12 related articles which were performed between 2001 and 2016 were selected for the final analysis [19–28]. All were cross-sectional studies and conducted in a variety of countries; 3 in the US [19, 21, 28], 2 in the UK [25, 27], 2 in Netherlands [23, 26], 1 each in Greece [22], Italy [20], and France [24]. The studies varied in their QoL measurement tools; 7 of them used SF-36 [19–21, 24, 25, 27, 28], 1 each used CHQ-PF50 [22], ITQOL [23], and CHQ [26].

Five studies included patients with NF1 [19, 20, 23, 24, 26], four studies included patients with NF2 [21, 22, 25, 27] and one study included both types of NF [28]. Studies on patients with schwannomatosis were very rare and their data could not be analyzed, so these studies were excluded from the study. These studies included a total of 642 patients with NF1, 268 patients with NF2. Also, the included studies consisted of six studies in adults [19, 20, 24, 25, 27, 28], three in children [22, 23, 26] and one study in both of them [21]. Considering all the included studies, the total number of participants was 7314 individuals containing 910 NF patients (782 adults and 128 children) and 6404 healthy subjects (5879 adults and 525 children), respectively. Table 2 summarizes the characteristics of the eligible studies. The outcomes evaluated included the total mean scores of physical function in 10 studies [19–28], physical role in nine studies [19–22, 24–28], bodily pain in ten studies [19–28], vitality in eight studies [19–22, 24, 25, 27, 28], mental health in eight studies [19–21, 24–28], emotional role in seven studies [19, 21, 24–28], social functioning in six studies [19, 21, 24, 25, 27, 28], general health in nine studies [19, 21–28] and family cohesion in three studies [22, 23, 26].

**Table 2:** Study characteristics

First author (references)	Year	Country	Age (M ± SD)	Number participant		QoL measure	Type of NF/group	Physical function case/control (M ± SD)	Role physical	Bodily pain	Vitality	Mental health	Role emotiona	Social functioning	General health	Family cohesion
				Case	Control											
Page. PZ [19]	2006	United States	43 ± 11.7	166	154	SF-36	1/Adult	78 ± 26 84 ± 22	70 ± 41 84 ± 22	63 ± 27 77 ± 23	47 ± 25 59 ± 21	63 ± 19 74 ± 23	70 ± 38 84 ± 22	74 ± 25 84 ± 22	55 ± 25 72 ± 23	–
Kodra. Y [20]	2009	Italy	37.7 ± 122	129	1909	SF-36	1/Adult	79 ± 26 86 ± 23	75 ± 34 80 ± 35	69 ± 27 75 ± 25	60 ± 20 63 ± 22	64 ± 21 67 ± 20				
Merker. VL [21]	2016	United States	13	8		SF-36	2/Children	54/8 52/7	53/6 52/3	56 50/9	53/5 50/3	49/9 49/4	52/7 51/2	49 50/6	49/8 51/2	
Vardarinos. A [22]	2009	Greece	10/1 ± 3	43	61	CHQ–PF50	2/Children	94/2 ± 16/7 94/6 ± 7/3	91/1 ± 22/8 97 ± 15/2	84/4 ± 19/5 94/9 ± 10/3	77/7 ± 18 83/1 ± 14/1				63/7 ± 19/4 79/8 ± 13/9	76/4 ± 22 83/1 ± 15/3
Ostenbrink. R [23]	2007	Netherlands	4 ± 1/5	34	410	IT-QOL	1/children	88/9 ± 20/3 97/2 ± 9/8		86 ± 15/9 83/8 ± 16/8					65/5 ± 16/5 79 ± 14/5	80/1 ± 22/1 75/3 ± 18/8
Wolkenstein. P [24]	2001	France		128	3656	SF-36	1/adult	76/8 ± 26/4 84/5 ± 21/1	72/8 ± 39/1 81/3 ± 32/2	65/3 ± 29/6 73/5 ± 23/7	49/7 ± 21/3 60/1 ± 18/1	56/4 ± 22 68/5 ± 17/6	69/4 ± 39/4 82/2 ± 32/2	70/4 ± 25/7 81/6 ± 21/4	58/4 ± 23 69/2 ± 18/6	
Hornigold. RE [25]	2015	UK	44	61	30	SF-36	2/Adult	57/4 ± 32/9 87/99 ± 19/65	51/5 ± 45/3 87/17 ± 22/01	67/3 ± 29 78/8 ± 23/01	49/2 ± 27/5 58/04 ± 19/6	61/84 ± 26/7 71/92 ± 18/15	59/3 ± 45/8 85/75 ± 21/18	64/7 ± 33/3 82/77 ± 23/24	48/7 ± 31/1 71/06 ± 20/43	
Krab. LC [26]	2008	Netherlands	12/2 ± 2/5	43	54	CHQ	1/children	95/7 ± 8/9 96/8 ± 5/4	96/1 ± 11/3 96/5 ± 11/6	71/4 ± 27/5 78/2 ± 19/5		79/8 ± 12/8 78/2 ± 13	92 ± 14/4 92/3 ± 16/8		72/7 ± 16/2 74/6 ± 15/9	75/6 ± 26/7 75/7 ± 23/1
Hornigold. RE [27]	2012	UK	41	50	30	SF-36	2/Adult	57/4 ± 33/9 84/25 ± 29/19	51/5 ± 46/53 83/85 ± 23/89	67/3 ± 29/94 81/1 ± 17/05	49/2 ± 28/4 62/6 ± 15/38	61/84 ± 29/16 74/5 ± 12/02	59/3 ± 47/23 82/65 ± 13/28	64/7 ± 33/91 88/2 ± 11/74	48/7 ± 31/74 73/05 ± 14/4	
Merker. VL [28]	2014	United state	38/7 ± 14/5 39/7 ± 29/7	142/53	50/50	SF-36	1,2/Adult	49/2 46/1 49/2 49/2	48 44/5 46/6 46/6	50/9 50/3 46/1 46/1	50/3 47/8 49/1 49/1	46/5 48 49/2 49/2	46/2 47/4 47/9 47/9	48/7 45 48/1 48/1	48/6 42/8 47/5 47/5	

### Statically analysis

Table 3 presents the total mean scores of sub-scales of QoL (SEM) using meta-analysis of data extracted from reviewed studies. We also compared mean scores [standard mean difference (SMD)] of sub-scales of QoL in subjects with and without NF to determine the association between NF and QoL (Table 3).

**Table 3:** Scores of sub-scales of QoL using random effect meta-analysis of data from reviewed studies

Sub-scales of QoL	Number of studies	SEM estimates (CI%95)		SMD (CI%95)	Heterogeneity index I^2^ (%)	*P* value
		NF	Control			
Physical Function	8	78.88 (70.19–87.58)	89.76 (84.91–94.61)	- 43 (−61, −24)	68.6	0.002
Role Physical	8	69.66 (55.09–84.23)	80.93 (71.65–90.22)	− 0.28 (− 0.51, − 0.06)	77.7	0.000
Bodily pain	9	71.74 (65.22–78.25)	80.40 (75.41–85.40)	− 0.35 (− 0.52,−0.19)	58.8	0.017
Vitality	6	50.06 (44.82–55.30)	57.37 (53.48–61.26)	− 0.42 (− 0.63, −0.21);	70.3	0.009
Mental health	8	63.83 (56.67–70.99)	69.57 (65.20–73.94)	− 0.37 (− 0.59, −0.15)	76.3	0.000
Role emotional	7	65.72 (51.91–79.53)	77.07 (69.37–84.76)	−0.44 (− 0.57, −0.32	26.0	0.000
Social functioning	6	64.32 (54.14–74.51)	75.32 (67.49–83.16)	−0.46 (− 0.57, −0.36)	14.3	0.000
General health	8	57.31 (51.77–62.86)	68.97 (63.88–74.06)	−0.65 (− 0.82, −0.47)	65.5	0.005
Family cohesion	3	77.38 (73.19–81.57)	78.06 (72.62–83.50)	−0.03 (− 0.39, 0.03)	62.1	0.072

**Fig. 2:**
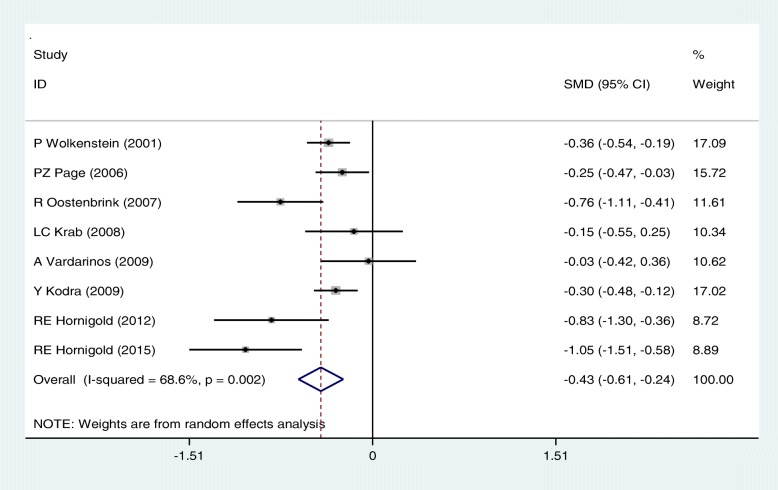
Meta-analysis of the association of Physical function with QoL in NF patients. Square represents effect estimate of individual studies with more than 95% confidence intervals with the size of squares proportional to the weight assigned to the study in the meta-analysis. In this chart, studies are stored in order of the year of publication and author’s names, based on a random effects model

As it is seen, ten studies were included for SEM estimates of physical function; the mean scores of physical function were significantly lower in people with neurofibromatosis disease: SEM estimates were 78.88(95%CI: 70.19–87.58) in the case group and 89.76(95%CI: 84.91–94.61) in control group respectively. The present meta-analysis with a random effects model showed a significant statistical difference in the Physical function status in NF patients compared with controls [SMD: - 43(95%CI: − 61, − 24); *P* < 0.05; I^2^ = 68.6%] (Fig. 2).

**Fig. 3:**
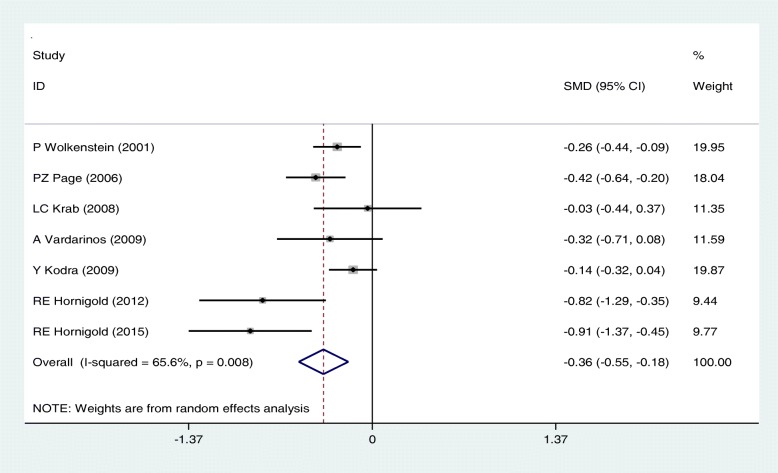
Meta-analysis of the association of Role physical with QoL in NF patients. Square represents effect estimate of individual studies with more than 95% confidence intervals with the size of squares proportional to the weight assigned to the study in the meta-analysis. In this chart, studies are stored in order of the year of publication and author’s names, based on a random effects model

Data from included studies were analyzed in a random-effects model to compare the role physical role in two groups; accordingly, SEM estimates in case and control group were 69.66(95%CI: 55.09–84.23) and 80.93(95%CI: 71.65–90.22) respectively and there was a significant difference. The SMD in the scores of the two groups were − 0.28[95%CI: − 0.51, − 0.06; I2 = 77.7%, *P* < 0.05: Fig. 3].

There was a significant difference in the the mean scores of Bodily pain in NF patients and controls [SMD: -0.29(95%CI: − 0.48,-0.10); I2 = 70.6%, *P* < 0.05]; The SEM estimates were 69.31 [95% CI: 62.01–76.61; *P* = 0.008] in NF group and 76.22 [95% CI: 70.14–82.30; *P* < 0.001] in the control group.

Our meta-analysis based on random-effects mode showed a significant difference in the mean scores of Vitality, Mental and General health in NF patients compared with controls; the SMD was − 0.42 [(95%CI: − 0.63,-0.21); I2 = 70.3%, *P* < 0.05], − 0.37[(95%CI: − 0.59,-0.15); I2 = 76.3%, *P* < 0.05] and − 0.65 [(95%CI: − 0.82,-0.47); I^2^ = 65.5%, *P* < 0.05] respectively.

Also, there was significant statistical difference in the the mean scores of Emotional role and Social functioning between NF patient and healthy subjects with a fixed effects model; the SMD was: -0.44 (95%CI: − 0.57,-0.32; I^2^ = 26.3%, *P* < 0.05] and [SMD: -0.46(95%CI: − 0.57,-0.36); I^2^ = 14.3%, *P* < 0.05].

As seen in Table 3, NF patients and control group had no significant differences in the mean scores of Family cohesion; SEM estimates were 77.38 (95%CI: 73.19–81.57) in the NF group and 78.06 (95%CI: 72.62–83.50) in the control group, respectively. The SMD in the scores of the two groups was − 0.03 [95% CI: − 0.39, 0.03; I2 = 62.1%, *P* = 0.072].

### Subgroup analyses

Due to sever heterogeneity between studies, we conducted subgroup analyses to minimize heterogeneity among various studies.

We chose to analyze the two neurofibromatoses (NF1 and NF2) together that are two different diseases in initial analysis because of the low number of obtained studies and data; also, in the second step and sub-group analysis, we have done a subgroup analysis on the basis of type of disease (NF1 and NF2). In the current study, the effect of neurofibromatosis on quality of life was examined by type of NF (NF1 and NF2); six studies were conducted in neurofibromatosis type 1 and sex in neurofibromatosis type 2. We found a significant difference in all sub-scales of QoL in patients with NF1compared with normal population. Also, the results of meta-analysis showed Type 2 neurofibromatosis negatively affected all aspects of quality of life (Table 4).

**Table 4:** The results of subgroup analyses

Sub-scales of QoL	Type questionnaire	Type NF	Group
Physical Function	SF-36:-0.46 (− 0.68, − 0.25) CHQ:-0.09 (− 0.37, 0.19) ITQOL:-0.76 (−1.11, − 0.41)	1: − 0.35 (− 0.49, − 0.20) 2:-0.63 (− 1.26, 0.01)	Children:-0.32 (−0.78, 0.13) Adult: − 0.46 (− 0.68, − 0.28)
Role Physical	SF-36:-0.43 (− 0.66, − 0.20) CHQ: − 0.18 (− 0.46, 0.10)	1: −0.24 (− 0.38, − 0.10) 2: − 0.66 (− 1.04, − 0.28)	Children: − 0.18 (− 0.46, 0.10) Adult: − 0.43 (− 0.66, − 0.20)
Bodily pain	SF-36: −0.38 (− 0.52, − 0.23) CHQ: − 0.50 (− 0.91, − 0.09) ITQOL: 0.13 (− 0.22, 0.48)	1: −0.28 (− 0.47, − 0.09) 2: − 0.57 (− 0.82, − 0.32)	Children:-0.28 (− 0.77, 0.21) Adult:-0.30 (− 0.52, − 0.09)
Vitality	SF-36: −0.42 (− 0.63, − 0.21)	1: −0.41 (− 0.69, − 0.12) 2: − 0.45 (− 0.76, − 0.13)	Children: Adult: − 0.42 (− 0.63, − 0.21)
Mental health	SF-36:-0.35 (−0.62, − 0.08) CHQ:-0.11 (− 0.57, 0.35)	1: −0.33 (− 0.66, − 0.01) 2: − 0.42 (− 0.66, − 0.17)	Children: − 0.11 (− 0.57, 0.35) Adult: − 0.45 (− 0.70, − 0.21)
Role emotional	SF-36: −0.47 (− 0.58, − 0.37) CHQ: − 0.02 (− 0.42, 0.38)	1: −0.41 (− 0.55, − 0.26) 2: − 0.64 (− 0.96, − 0.32)	Children: − 0.02 (− 0.42, 0.38) Adult: − 0.47 (− 0.58, − 0.37)
Social functioning	SF-36: −0.47 (− 0.59, − 0.35)	1: −0.44 (− 0.55, − 0.33) 2: − 0.71 (− 1.04, − 0.39)	Adult: −0.47 (− 0.59, − 0.35)
General health	SF-36: − 0.61 (− 0.77, − 0.45) CHQ: − 0.55 (− 1.40, 0.30) ITQOL: − 0.92 (− 1.28, − 0.57)	1: − 0.55 (− 0.75, − 0.35) 2: − 0.90 (− 1.16, − 0.65)	Children: − 0.68 (− 1.21, − 0.14) Adult:-0.61 (− 0.77, − 0.45)
Family cohesion	CHQ: −0.18 (− 0.54, 0.17) ITQOL: 0.25 (− 0.10, 0.60)	1: 0.14 (− 0.12, 0.41) 2: − 0.36 (− 0.76, 0.03)	Children: − 0.03 (− 0.39, 0.33)

A subgroup meta-analysis was also performed by age group (children and adult). We included the patients from adolescents to adults, this would add the bias of the statistics. Adolescents and adults share different sub-scales of QoL, especially in “Mental health” and “Social functioning”. The scale of “Mental health” and “Social functioning” in adolescents cannot be used to assess the actual condition of adults and vice versa. Researchers of the obtained studies have focused on the topic and have used standard and appropriate tools for any age group. We have also done a subgroup analysis on the basis of age group that minimizes this bias and solves the problem. Eight studies were performed in adults and the results of meta-analysis showed that neurofibromatosis diseases affected all aspects of quality of life in adult patients. Among reviewed studies, 4 were conducted in children. Our meta-analysis showed that neurofibromatosis did not negatively affect various aspects of quality of life in children (Table 4).

As can be seen in Table 4, the validated tools used to measure QoL in reviewed studies were SF-36, CHQ-PF50, and ITQOL. So, we conducted subgroup analysis by type of questionnaire; the result showed that NF had negative effects on all sub-scales of QoL if the study used a SF-36 questionnaire.

**Fig. 4:**
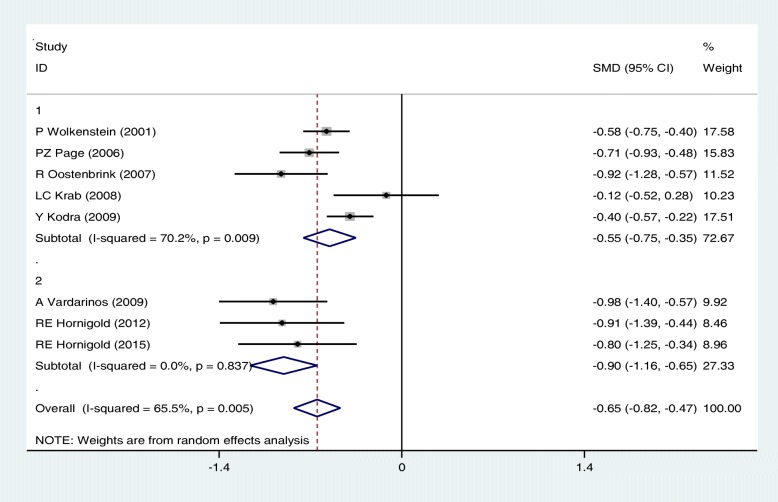
Meta-analysis of the association of General health with QoL in NF patients based on type of NF(1: NF1, 2:NF2). Square represents effect estimate of individual studies with more than 95% confidence intervals with the size of squares proportional to the weight assigned to the study in the meta-analysis. In this chart, studies are stored in order of the year of publication and author’s names, based on a Random Effects Model

**Fig. 5:**
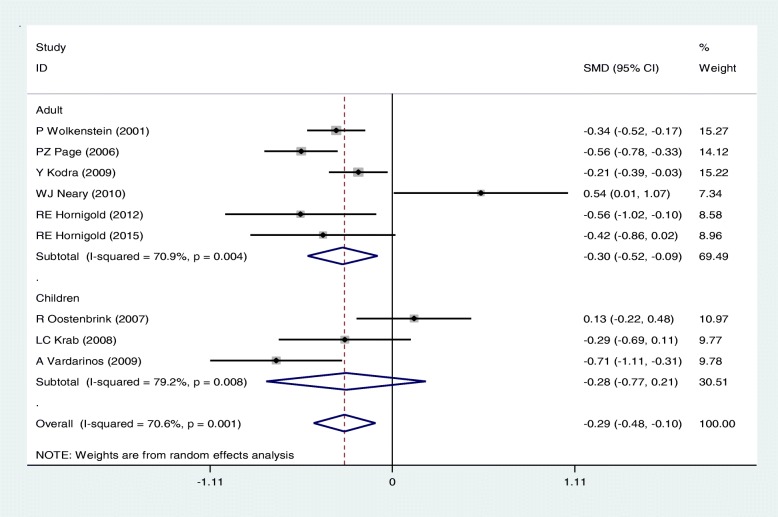
Meta-analysis of the association of Bodily pain with QoL in NF patients based on age group. Square represents effect estimate of individual studies with more than 95% confidence intervals with the size of squares proportional to the weight assigned to the study in the meta-analysis. In this chart, studies are stored in order of the year of publication and author’s names, based on a fixed effects model

**Fig. 6:**
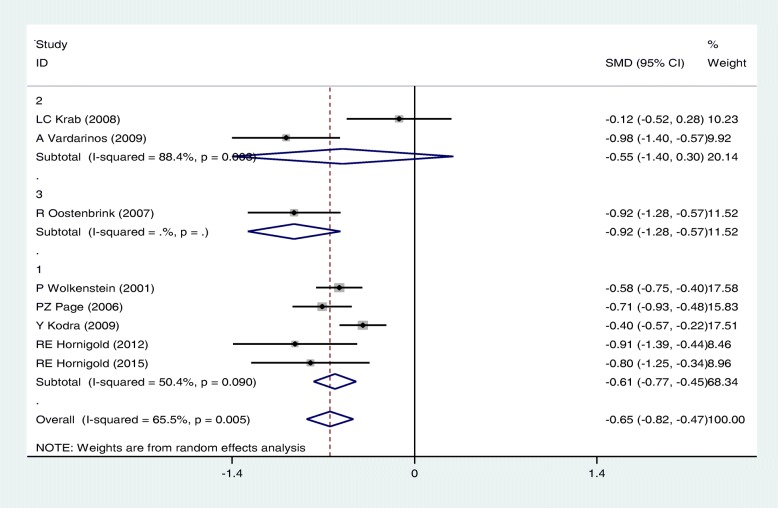
Meta-analysis of the association of General health with QoL in NF patients based on type of questionnaire (1: SF-36, 2: CHQ, 3: ITQOL). Square represents effect estimate of individual studies with more than 95% confidence intervals with the size of squares proportional to the weight assigned to the study in the meta-analysis. In this chart, studies are stored in order of the year of publication and author’s names, based on a Random effects model

In general, subgroup analysis showed that the results concerning the association of NF and QoL may vary with age group and type of questionnaire, but do not change with type of NF. Figures 4, 5 and 6 present the forest plots of the association of some sub-scales of QoL with NF based on type of NF, age group and type of questionnaire, respectively.

### Publication bias

**Fig. 7:**
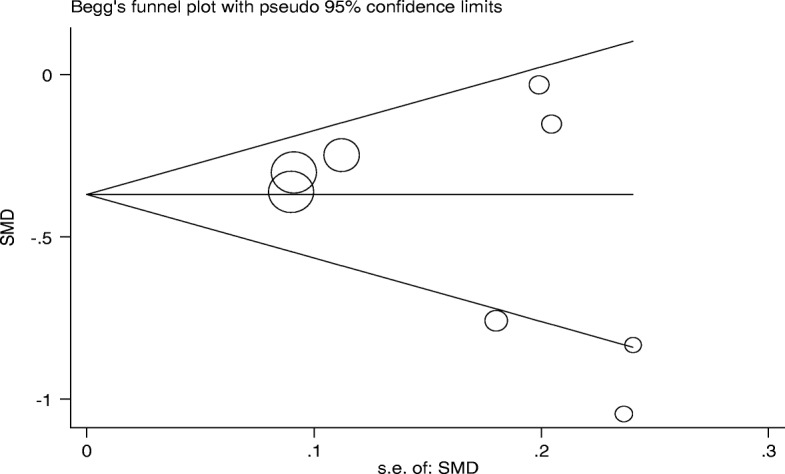
Begg’s funnel plot for publication bias in the risk difference (RD) analysis. The diameter of each circle represents the weight in the meta-analysis

Publication bias was detected by drawing Beggs funnel plot in the meta-analysis. This diagram showed that there was no significant publication bias (*p* = 0.24). This means that it is possible that studies with the negative results have not been published (Fig. 7).

## Discussion

This study systematically reviews the quality of life of neurofibromatosis patients in children and adults. To assess the quality of life scales, the CHQ-PF50, and ITQOL, SF-36 questionnaires, which had similar scales, were examined. In all reported studies, neurofibromatosis patients had lower quality of life than controls.

In all sub-scales studied: There was a meaningful relationship between the reduction of QoL criteria and fibromatosis light disease, except for the scale of Family cohesion. As it is seen in Table 2, the scale of Family cohesion was assessed in 3 studies in children. Out of these studies, in one study, the mean of the family cohesion scale in the neurofibromatosis patients group was lower than that in the control group, it was equal in two groups in one study and was higher in neurofibromatosis patients than control group in another study. The overall estimation in this study showed that Type 1 neurofibromatosis did not affect all aspects of quality of life in children. Finally, there was no significant relationship between QoL and Neurofibromatosis.

The present study was a meta-analysis of previous conducted studies on the quality of life of patients affected by NF. This systematic review and meta-analysis including 910 individuals from 12 studies was conducted to comprehensively review this literature and investigate quantitatively the QoL in patients with NF. All studies received high quality ratings. The results of our meta-analysis showed the general QoL has decreased significantly in people with neurofibramatosis disease compared to the general population. Decreased QoL was observed in physical, mental, and social domains. To our knowledge, there are no other meta-analyses similar to ours to compare the results. However, most of our findings were consistent with the findings of studies reported previously that indicated NF could have profound effects on the QoL of patients in a variety of domains [31, 32]. A 2013 systematic review summarizes the results of eight studies on the quality of life among adult patients with NF [30]; most of these studies reported lower quality of life on all subscales (physical functioning, role physical, bodily pain, general health, vitality, social functioning, role emotional, and mental health) in patients with NF when compared to the general population [30]. This review also indicated visibility and disease severity were strong predictors of skin-specific quality of life in NF patients [30]. Another systematic review of seven original studies in 2015 among children and adolescents with NF reported that these patients have lower general QoL compared to population norms in at least some subdomains [31]; these subscales in patients with NF1 (7–16 years old) included domains of motor, cognitive, social functioning, and emotional functioning [34]. In preschool children with NF1, they were physical functioning, growth and development, general behavior, general health perceptions, parent emotional impact, and parental time impact [23]. However, the bodily pain QoL subscale was the only domain in a study with patients aged 10–14 years [26]. Also, skin-specific QoL was lowered in children with NF1 ages 8–16 [35].

Our study reported that QoL is negatively affected by all subdomain physical functions, bodily pain, mental health, social function and general health in NF patients. These results are consistent with previous previous studies, which showed individuals with NF experience decreased QoL in all subdomains of QoL [4, 9, 19, 20, 23–25]. The studies have outlined a framework that included five domains reflecting the most important concerns: physical function impact, bodily pain, social functioning, sigma, and emotional distress [36]. A review study on functioning, disability and health in children with neurofibromatosis type 1 provided a beginning understanding of some QoL concerns from the perspectives of youth and their families [37]. These QoL concerns were related to specific symptoms or complications of NF1, the variability of manifestations of the disorder, its uncertain course, the visibility of some signs and symptoms, and the presence of comorbidities [37]. A study in 2016 using a qualitative approach explored some concerns of patients with NF1 regarding the QoL across the lifespan [38]. In this study the patients and their families were most concerned about physical functioning, pain, appearance/disfigurement, social activity/role participation, stigma, anxiety and social relationships [38]. Another study conducted based on semi-structured interviews revealed additional areas of concern including cognitive functioning, family impact, and treatment burden [36]. Our findings and these results suggest that NF is a broad spectrum disease that represents a considerable burden for patients, affecting all aspects of life.

We conducted a subgroup analysis according to age group; our meta-analysis showed that QoL in all subdomains (physical function, mental and emotional status, social functioning status and general health) has decreased significantly in adult NF patients. However, the reverse was true for affected children; we found no significantly lower QoL for children with NF in most subdomains when compared with children’s norms [data not shown]. There was some strong evidence suggesting that children affected by NF may have low-level QoL compared to general population [23, 24, 26], but our quantitative meta-analysis did not confirm this result. One possible cause of the observed difference in findings may be due to limited studies and/or poor methodology, heterogeneity of measurements and limitations of the available data. Few studies assessed the role of subdomains of QoL in children with NF, also most of them were largely focused on disease-related variables and did not explore association’s subdomains with QOL; thus results described in this subgroup were partly inconclusive. It was acknowledged that evaluating symptoms and complications related to NF in adult patients provides a more informative and reliable evaluation than those in children [26]. On the other hand, the observation illustrates that NF is a chronic disease evolving over time, in other words, during childhood some symptoms and complications related to NF may not appear, whereas some can become more prominent during adolescence and even during adulthood [39].

We found a meaningful relationship between the reduction of QoL criteria and NF due to the various physical, cognitive, and social complications. The most common complications of NF are behavioral problems and cognitive disorders [19, 21–25, 27]. It is important to note that the burden of NF is the link to its self-perception and NF represents an assault on the self-image that is correlated to its visibility; because NF1patients often have visible external tumors that affect on their appearance and these patients are more likely to develop negative self-concepts that are associated with stigma, psychiatric morbidity and social exclusion [30, 31, 40, 41].

Thus evaluation and treatment of symptoms and complications related to NF seems important in increasing the quality of life of NF patients. The results of such studies could help to implement beneficial strategies about QoL in this population. Socioeconomic status, sex, familial NF, NF severity and the presence of behavioral problems influence several QoL domains that can become targets of future clinical interventions aimed at improving QoL in NF patients [22]. Also, the factors related to QoL in patients with NF should be routinely assessed and a medical cure found.

This meta-analysis had several limitations: Firstly, there is a relative lack of high quality studies in patients with NF. Secondly, the studies varied in their QoL measurement tools and demographic features of the NF population (age, severity, complications) that could have influenced the results. Thirdly, we were unable to evaluate the impact of some important factors such as socioeconomic psychosocial interventions for improving quality of life in this population.

### Limitations

Status, sex, familial NF1, NF1 severity and the presence of behavioral problems that influenced several QoL domains because of insufficient data were all limitations of this study. Furthermore, some studies associated with QoL in NF patients were not accessible. Finally, in a meta-analysis of published studies, publication bias is an inevitable problem.

## Conclusion

This meta-analysis shows that NF had a negative effect on the general QoL and individuals with NF experience decreased QoL in physical function, bodily pain, mental health, social function and general health. Data from our study also demonstrated that QoL is markedly negatively affected by all subdomains in adult NF patients. The results somewhat differed between adults versus children; we found no significantly lower QoL for children with NF in most subdomains when compared with children’s norms; thus the data related to QoL in children provided inconclusive results due to low number of studies, heterogeneity of measurement tools, limitations of the available data and changes in QoL subdomains over time. In conclusion, data from this study indicate NF is a disease that interacts to impact well-being and disability, because it can impair physical, emotional, cognitive and social functioning. Based on our findings, strategies and programs for prevention of many symptoms and complications related to NF are needed, and the investment of resources and time are essential to helping these patients achieve a higher quality of life. Clinicians should provide comprehensive care for individuals with NF. We suggest applying medical cure and psychosocial interventions for improving quality of life in this subgroup.

## Data Availability

All data generated or analyzed during this study are included in this published article.

## Abbreviations

HRQoL: Health related quality of life
NF: Neurofibromatoses
NOS: Newcastle–Ottawa Scale
QoL: Quality of life
